# Diaphragm Position on Chest Radiograph to Estimate Lung Volume in Neonates

**DOI:** 10.1001/jamapediatrics.2025.2108

**Published:** 2025-07-21

**Authors:** Sophia I. Dahm, Arun Sett, Emma F. Gunn, Fiona Ramanauskas, Richard Hall, David Stewart, Sienna Koeppenkastrop, Kieran McKenna, Rebecca E. Gardiner, Padma Rao, David G. Tingay

**Affiliations:** 1 Neonatal Research, Murdoch Children’s Research Institute, Melbourne, Victoria, Australia; 2 Medical Workforce, Royal Children’s Hospital, Melbourne, Victoria, Australia; 3 Department of Paediatrics, The University of Melbourne, Victoria, Australia; 4 Department of Obstetrics, Gynaecology and Newborn Health, The University of Melbourne, Victoria, Australia; 5 Newborn Services, Joan Kirner Women’s and Children’s, Sunshine Hospital, Western Health, St Albans, Victoria, Australia; 6 Department of Medical Imaging, The Royal Children’s Hospital, Melbourne, Victoria, Australia; 7 Department of Neonatology, Royal Children’s Hospital, Melbourne, Victoria, Australia

## Abstract

**Importance:**

Using chest radiographs to guide lung aeration during respiratory support in infants is common practice and recommended in neonatal intensive care unit (NICU) guidelines, but this practice has never been validated.

**Objective:**

To describe the association between diaphragm position on chest radiograph in infants and aerated lung volume calculated from computed tomography (CT).

**Design, Setting, and Participants:**

This was a retrospective cross-sectional study conducted at a tertiary children’s hospital, the Royal Children’s Hospital, in Melbourne, Australia. Included were infants without congenital lung pathology who received a chest CT in the first 30 days after birth between July 9, 2012, to December 31, 2022; infant data were retrieved from the Royal Children’s Hospital Medical Imaging database. Study data were analyzed from December 2022 to September 2023.

**Exposure:**

Lung volume was calculated using CT semiautomated tissue segmentation and diaphragm position determined using a standardized definition. All investigators analyzing CTs were unaware of the chest radiograph measurements and vice versa.

**Main Outcomes and Measures:**

The primary outcome was the distribution and precision of total lung volume at each of the measured diaphragm positions (6th-11th posterior rib).

**Results:**

The imaging data of 218 infants (median [IQR] age, 11 [3-20] days old; mean [SD] age, 37.9 [1.9] weeks’ gestation at birth; 119 male [55%]) were analyzed. Infants had a mean (SD) weight of 3055 (584) g at scan, and 132 (61%) had a primary cardiac diagnosis. The number of posterior ribs representing diaphragm position ranged from 6 to 11. There was only a weak association between diaphragm position and lung volume (Kendall τ = 0.23; 95% CI, 0.16-0.31). A similar weak association was observed by hemithorax (left, Kendall τ = 0.25; 95% CI, 0.15-0.34; right, Kendall τ = 0.21; 95% CI, 0.10-0.31), degree of consolidation (Kendall τ = 0.30; 95% CI, 0.21-0.38), apex-diaphragm distance (Kendall τ = 0.40; 95% CI, 0.28-0.51), and Hounsfield unit values (Kendall τ = −0.05; 95% CI, −0.15 to −0.06).

**Conclusions and Relevance:**

Results of this cross-sectional study suggest that despite long-standing acceptance in the NICU, the use of diaphragm position on chest radiograph lacked the precision required to assess aerated lung volume and guide clinical practice in infants.

## Key Points

**Question:**

Does the use of diaphragm position relative to posterior ribs on chest radiograph reliably estimate aerated lung volume in neonates?

**Findings:**

In this cross-sectional study that included 218 neonates, the association between diaphragm position and lung volume was weak.

**Meaning:**

Results suggest that the commonly used method of estimating lung volume via diaphragm position on chest radiograph lacks the precision required to guide clinical practice in neonates.

## Teaser

This cross-sectional study investigates if use of diaphragm position relative to posterior ribs on the chest radiograph of neonates reliably estimates aerated lung volume.

## Introduction

Accurate estimation of lung volume is crucial for safe and effective delivery of respiratory support in critical care.^1,2^ Measuring the diaphragm position through counting the number of ribs on chest radiographs is a simple method of estimating lung volume that has been used to guide respiratory support decisions for over a century.^3,4^ This is especially so in the neonatal intensive care unit (NICU).^5,6,7^ To our knowledge, only 1 small study^7^ has investigated the association between diaphragm position to absolute lung volume (using inert gas washout) in infants receiving mechanical ventilation and found no association. Therefore, although common clinically, the practice is without an evidence base.^8^

National and international guidelines recommend obtaining a chest radiograph to diagnose disease processes and estimate lung volume in infants needing respiratory support, especially during high-frequency ventilation.^6,9,10,11,12^ Values of 7 to 9 posterior ribs to define optimal aeration, fewer than 7 ribs hypoinflation, and more than 9 ribs hyperinflation are commonly quoted in guidelines.^5,6^

The unvalidated practice of measuring diaphragm position through rib numbers on a chest radiograph has likely evolved from the difficulties in reliably assessing lung volume at the bedside in infants. Accurate methods such as spirometry, plethysmography, and inert gas washout are too invasive, complex, or time consuming to be used in the NICU.^11^ Computed tomography (CT) is an accepted criterion standard method of assessing lung volume in all age groups and routinely used in critical care.^12^ CT systems are able to differentiate lung tissue and accurately compute lung volume using automated tissue density segmentation tools.^13^ This is calculated by evaluating a tissue area of interest as a proportion of its Hounsfield units (HU), a quantitative measurement of radiodensity. Although fully automated CT-derived lung volume measurements have been validated in the adult population, these automated calculations cannot be reliably performed on infant CT scans due to the lower radiation doses and resultant poorer image quality.^14^ An alternative method is to first manually delineate the areas of interest and then calculate lung volume using a semiautomated segmentation approach.^15^

The aim of the Calculation of Lung Inflation in Neonates (COLIN) study was to determine the association between the diaphragm position relative to the number of posterior ribs visualized on a chest radiograph equivalent (CT topogram) with aerated lung volume in infants. The primary outcome was the distribution and precision of total lung volume (measured as milliliter per kilogram of body weight) at each of the measured diaphragm positions (6th-11th posterior rib). Secondary outcomes included the distribution of right and left lung volume and HU at each measured diaphragm position, by indication for CT scan, respiratory support, gestation, and degree of consolidation. The apex-diaphragm distance was added as an a priori exploratory outcome.

## Methods

This study was approved by the Royal Children’s Hospital Melbourne Human Research Ethics Committee as a retrospective study and prospectively registered with the Australian New Zealand Clinical Trials Registry. As deidentified data were used, in accordance with local regulations, guardian consent was deemed not required by our Human Research and Ethics Committee. This study followed the Strengthening the Reporting of Observational Studies in Epidemiology (STROBE) reporting guidelines.

### Patient Population

Eligible infants were identified from the Royal Children’s Hospital Medical Imaging database. Infants who received a chest CT for any reason in the first 30 days after birth were eligible for inclusion. Infants were not included if there was a primary diagnosis of congenital lung pathology. Demographic data were extracted from the medical record for all included infants. Race and ethnicity information was not collected as it could not be accurately determined from the imaging dataset and was not considered to have an influence on the study aims or results. Where an infant had multiple CT scans, only the first CT was used.

### Image Reporting

**Figure 1.**
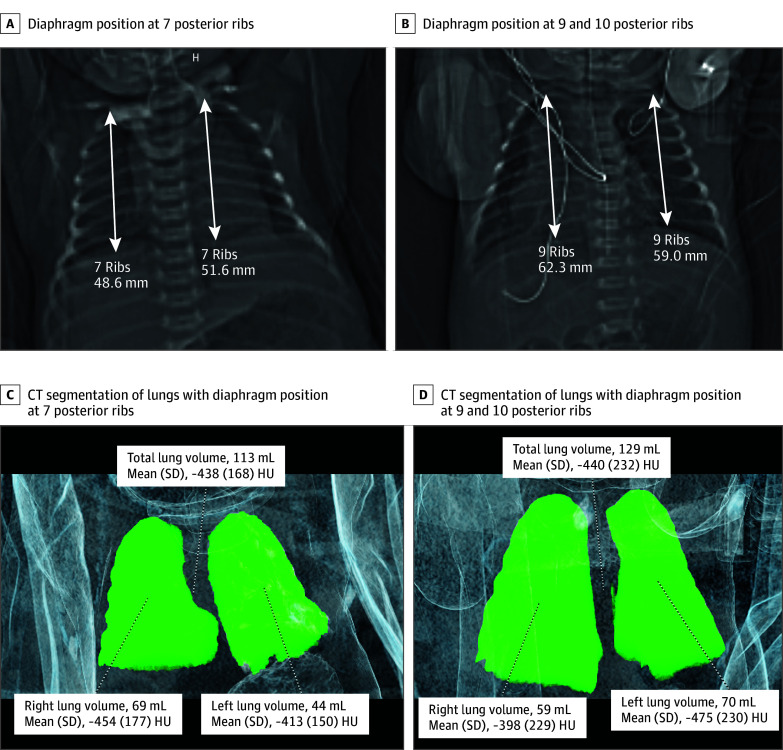
Chest Radiograph Equivalent for an Infant Chest radiograph equivalent for an infant with diaphragm position at 7 posterior ribs (A) and 9 and 10 posterior ribs (B) to summarize the association of diaphragm position with posterior rib. The diaphragm position was defined as the last posterior rib that completely intersects the diaphragm or had the diaphragm positioned entirely within the posterior rib. The rib above was used if no posterior rib intersected the diaphragm. When the diaphragm involved 2 posterior ribs but did not completely transect the lower rib, the upper rib was used if less than 50% of the intercostal space between ribs included lung fields and vice versa as seen in panel B (right chest) (eMethods and eFigure 1 in Supplement 1). Apex-diaphragm distance also shown. Total, left, and right lung volume and Hounsfield units (HU, a quantitative measurement of radiodensity) measurements from computed tomography segmentation of the lungs in each respective infant (C and D). Despite the different diaphragm positions, the absolute lung volume were similar.

A localizer image (scout chest topogram) is performed at the start of every CT scan to confirm the area for scanning. For the purposes of this study, the chest topogram (termed *chest radiograph equivalent* [CRE] for this study) was used to measure diaphragm position for the purpose of assessing the ribs and diaphragm. Although topograms have lower resolution, differences in image blur, beam hardening, and detector shape when compared with chest radiographs, they have been found to be comparable with chest radiographs for assessing ribs and identifying incidental lung pathology.^16,17^ As multiple definitions exist for the diaphragm position,^5,6,18^ in our study, the diaphragm position was defined by consensus of 3 neonatologists (1 with over 20 years’ clinical experience [D.G.T.], and 2 with more than 5 years’ experience [A.S. and D.S.]) and 1 pediatric radiologist (R.E.G.) using the available literature as detailed in Figure 1 and the eMethods and eFigure 1 in Supplement 1. Each neonatologist was randomly assigned a subset of CREs for assessment and blinded to the result of the CT scan. The left and right lung vertical apex-diaphragm distance (measured in millimeters) was also calculated (eFigure 2 in Supplement 1). To replicate clinical practice, each investigator also subjectively reported the presence of rotation, lordosis, quality of the image (good, acceptable, poor), and any subjective features of atelectasis/consolidation and hyperinflation.

Lung volume was calculated from included CT scans using the syngio.via (Siemens Healthcare GmbH) semiautomated tissue segmentation tool, as detailed in the eMethods in Supplement 1. Briefly, lung volume was calculated from each scan using the region growing (airway) tool and refined manually (eFigure 3 in Supplement 1). Lung volume calculations of the left, right, and total lung were standardized to weight (measured as milliliters per kilogram). In addition, HU measurements (maximum, minimum, mean, and SD) were calculated for all 3 lung regions. Finally, the presence of radiographic opacification of the parenchymal lung tissue (none, mild, moderate, or severe) was subjectively scored and termed *consolidation* with severe indicating minimal aeration of the lungs. The quality of each scan was subjectively coded as good, acceptable, or poor to categorize segmentation difficulty. All investigators analyzing CTs were masked to the CRE measurements.

Interobserver and intraobserver agreement of the CT semiautomated tissue segmentation tool and calculation of diaphragm position was first assessed with a prespecified group of 30 infants (eFigures 4-8 in Supplement 1). These infants were included in the final analysis.

CREs and CT images deemed of poor quality by their initial reviewer were reviewed by a second investigator assessed by the study team with consensus opinion determining inclusion. Specifically, if the lung regions were missing in more than 2 planes of the CT scan, or the diaphragm and/or complete chest wall boundaries were not visible in the CRE, the infant was excluded from analysis.

### Statistical Analysis

The primary outcome was the CT measurement of lung volume at each diaphragm position relative to posterior rib on CRE. Following statistical advice at study conception, a feasibility sample size of at least 200 CT scans was recommended to ensure an adequate distribution of measurements across all potential diaphragm position measurements (6 to 11 posterior ribs), anticipating an uneven distribution of data favoring 8 to 10 posterior ribs. Demographic data are presented as mean (SD) or median (IQR) depending on data distribution. Strength of association between variables is calculated using Kendall τ or Pearson *r* correlation analysis as appropriate (using R, version 4.4.1 [R Project for Statistical Computing]). A weak correlation coefficient was a priori defined as a Kendall τ or Pearson *r* value of less than 0.40; moderate, 0.40 to 0.69; and strong, greater than or equal to 0.70. Study data were analyzed from December 2022 to September 2023.

## Results

A total of 292 infant CT scans were available for analysis, with 40 scans excluded at initial screening due to repeated scans from the same infant (20 scans) or duplicate records (16 scans) (eResults and eFigure 9 in Supplement 1). Of the 252 included scans, 34 were then excluded for poor image quality (CT, 8; CRE, 11) or meeting further exclusion criteria (15 scans), resulting in a final study population of 218 infants (median [IQR] age, 11 [3-20] days old; mean [SD] age, 37.9 [1.9] weeks’ gestation at birth; 99 female [45%]; 119 male [55%]). Table 1 and the eResults and eTable in Supplement 1 describe the demographic data of the included infants. Infants had a mean (SD) weight of 3055 (584) g at scan, and infants were mostly born at term and had a cardiac indication (132 [61%] had a primary cardiac diagnosis) for CT. Phase of the respiratory cycle during the CT was only reported in 5 infants (2 inspiration, 3 expiration). Thus, the impact of gestational age, respiratory phase, and indication for CT was not analyzed.

**Table 1.** Characteristics of Included Infants (N = 218)

Characteristic	No. (%)^a^
Age at CT scan, median (IQR), d	11 (3-20)
Gestation, mean (SD), completed wk	37.9 (1.9)
Birth weight, g	3055 (584)
Weight at CT scan, mean (SD), g	3154 (633)
Sex	
Female	99 (45)
Male	119 (55)
Primary diagnosis	
Preoperative cardiac disease	131 (60)
Postoperative cardiac disease	74 (34)
Parenchymal lung disease	2 (1)
Other^b^	11 (5)
Comorbidities	
Preterm birth <30 wk of gestation	1 (0.5)
Preterm birth 30-36 wk of gestation	22 (10)
Cyanotic cardiac disease	36 (17)
Noncyanotic cardiac disease	14 (6)
Extracorporeal membrane oxygenation	15 (7)
Other^b^	50 (23)
Indication for CT	
Preoperative cardiac planning	73 (33)
Postoperative cardiac planning	68 (31)
Diagnostic (cardiac disease)	45 (21)
Diagnostic (lung disease)	7 (3)
Investigation of undifferentiated hypoxia	14 (6)
Other^b^	11 (5)

Abbreviation: CT, computed tomography.

^a^ Numbers listed as No. (%) unless otherwise indicated.

^b^ Details available in the eResults in Supplement 1.

The characteristics and details of CRE and CT scans are shown in Table 2. The number of posterior ribs on CRE ranged from 6 to 11, with 95% of posterior rib measurements between 8 to 10 ribs. A total of 5 CREs and 32 CT scans were assessed as being of poor image quality and/or exposure. Overdistention was present in 29 CREs (13%) and atelectasis in 22 CREs (10%). Parenchymal opacification was present in 133 CT scans (61%), with 69 (32%) classified as moderate or severe. In 119 CREs (55%), the diaphragm position (posterior rib number) was equal for the left and right hemithorax, with the left diaphragm position having a greater rib number in 77 infants (35%) and right diaphragm having a greater rib number in 22 infants (10%). There was poor to moderate interobserver agreement of diaphragm position on CRE between the 3 individual observers (Cohen κ coefficient, 0.20-0.67) (eFigure 7 in Supplement 1).

**Table 2.** Chest Radiograph Equivalent and Computed Tomography (CT) Features^a^

Characteristic	No. (%)
Respiratory support during CT scan	
Spontaneously breathing	131 (60)
Mechanical ventilation via an ETT	77 (35)
Unknown	10 (5)
Image quality	
CRE	
Good	162 (74)
Acceptable	51 (23)
Poor	5 (2)
CT	
Good	127 (58)
Acceptable	59 (27)
Poor	32 (15)
Chest radiograph features	
Lordosis	15 (7)
Rotation	103 (47)
Both clavicles fully visible	207 (59)
Atelectasis	22 (10)
Features of overdistension^b^	29 (13)
No. of posterior ribs (n = 436)	
6	1 (0.2)
7	19 (4)
8	140 (32)
9	212 (49)
10	60 (14)
11	4 (1)
Diaphragm position (No. of ribs)	
Left equal to right	119 (55)
Left greater than right	77 (35)
Left less than right	22 (10)
CT features	
Parenchymal opacification or consolidation	
Mild	64 (29)
Moderate	43 (20)
Severe	26 (12)
Chest drains present	26 (12)
CT contrast used	210 (96)
Pneumothorax present	5 (2)
Incomplete lung fields on CT	7 (3)

Abbreviations: CT, computed tomography; CRE, chest radiograph equivalent; ETT, endotracheal tube.

^a^ CRE and CT features were classified by the reviewer.

^b^ Overdistension on CRE was defined as flattened diaphragm, mediastinal or cardiac narrowing, rib bulging, or subjectively overdistended lung fields.

**Figure 2.**
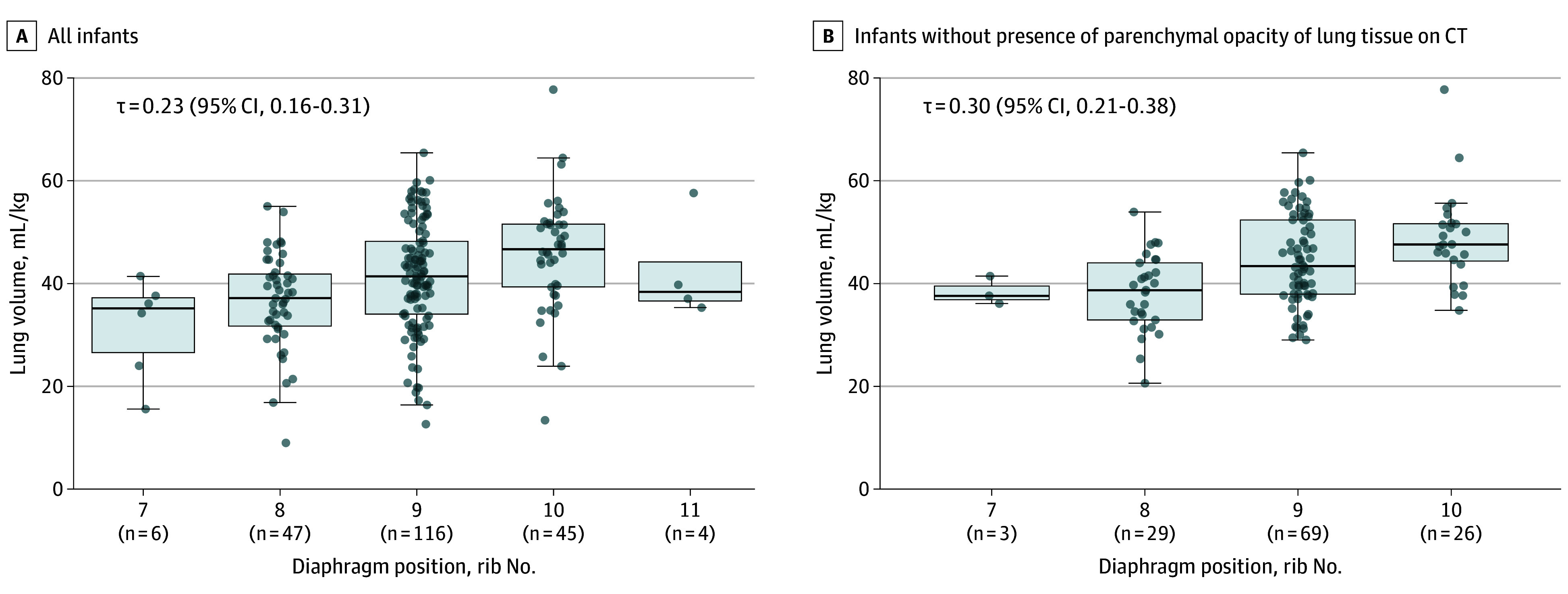
Association Between Total Lung Volume and Diaphragm Position Defined by Posterior Rib Association between total lung volume and diaphragm position defined by posterior rib (rib number) for all infants (A) and in infants without presence of parenchymal opacity of lung tissue on computed tomography (CT; no consolidation; B). Where left and right diaphragm position differed, the greater posterior rib number was selected for comparison. Boxes represent IQR and horizontal line median. Whiskers represent 1.5 × IQR. Individual data points shown as gray circles. Diaphragm position was assessed by 3 neonatologists (A.S., n = 70; D.S., n = 39; D.G.T., n = 109). τ indicates Kendall τ.

**Figure 3.**
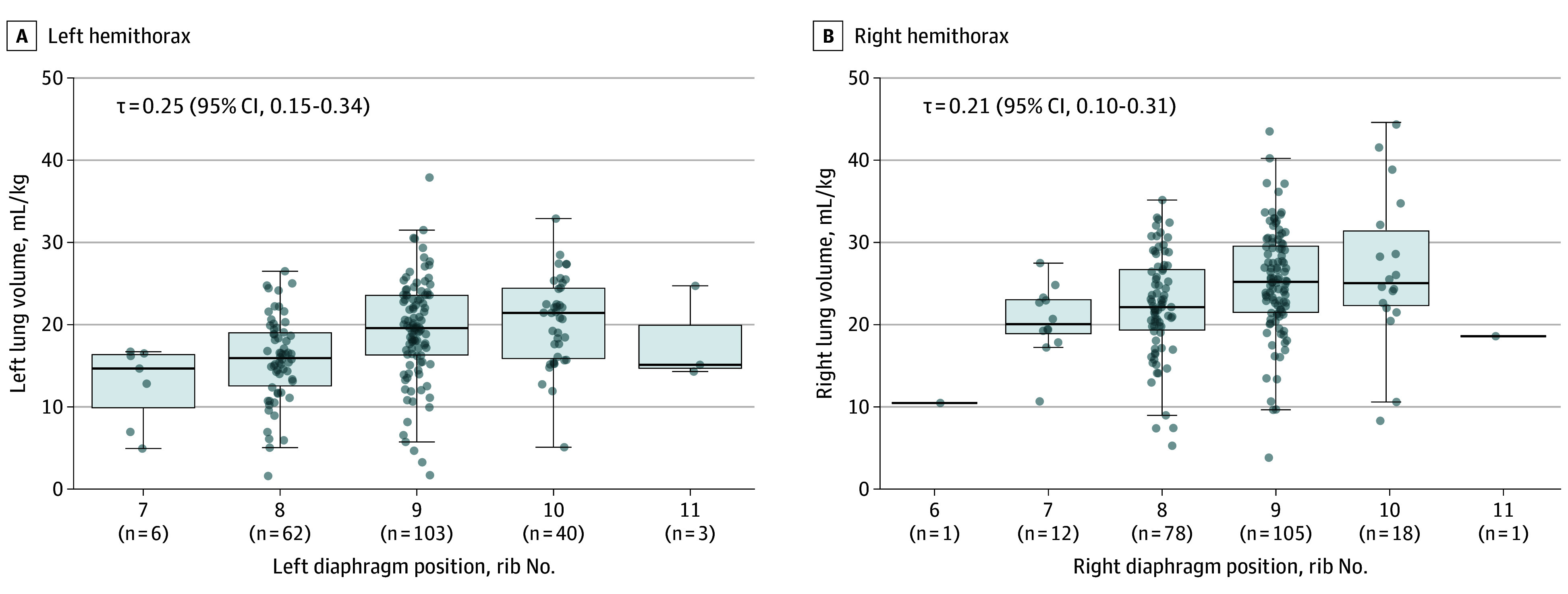
Association Between Lung Volume and Diaphragm Position in the Left and Right Hemithorax Association between lung volume and diaphragm position (posterior rib number) in the left (A) and right (B) hemithorax. Boxes represent IQR and horizontal line median. Whiskers represent 1.5 × IQR. Individual data points shown as gray circles. τ indicates Kendall τ.

Mean total lung volume increased with greater posterior rib count diaphragm position, however, the association was weak with large variability and distribution of lung volumes at each rib number (using the greatest rib number when discordant between hemithoraces; Kendall τ = 0.23; 95% CI, 0.16-0.31) (Figure 2A). The inclusion of only infants with consolidation-free images did not meaningfully improve the association (Kendall τ = 0.30; 95% CI, 0.21-0.38) (Figure 2B), nor did assessing the right and left hemithorax in isolation (left, Kendall τ ** = ** 0.25; 95% CI, 0.15-0.34 and right, Kendall τ = 0.21; 95% CI, 0.10-0.31) (Figure 3). The association was similar using the smaller rib number when discordant (Kendall τ = 0.24; 95% CI, 0.17-0.32) (eFigure 10 in Supplement 1) or absolute lung volume not referenced to body weight (eFigure 11 in Supplement 1). There was no difference in the association when comparing CREs identified as overdistended (eFigure 12 in Supplement 1) or atelectatic (eFigure 13 in Supplement 1).

The association between diaphragm position and total mean HU, the average aeration within the lungs (Kendall τ = −0.05; 95% CI, −0.15 to 0.06) and total SD HU, the variability of aeration throughout the lungs (Kendall τ = −0.01; 95% CI, −0.01 to 0.12) was weak (eFigure 14 in Supplement 1). This weak association was unchanged using lowest diaphragm position (if discordant) and left and right hemithorax (eFigure 15 in Supplement 1). The average apex-diaphragm vertical distances also poorly correlated with lung volume (eFigure 16 in Supplement 1).

## Discussion

The long-standing use of diaphragm position on chest radiographs to estimate lung volume in infants has been extrapolated from use in adults and is largely unvalidated in both populations.^3^ Despite the lack of evidence, this specific radiographic estimate of lung volume is frequently used to titrate respiratory support settings in NICU patients. To our knowledge, this was the first large study to assess the association between radiographic diaphragm position and lung volume in any ICU population. We demonstrated that there was only a weak association between posterior rib number and lung volume. Together, this suggests that using radiographic diaphragm position lacks the precision and accuracy to be used to guide respiratory support in infants.

The diaphragm position in relation to the posterior ribs is the most used chest radiograph measurement to assess lung volume.^6,9,19^ To date and to our knowledge, only 1 small study^7^ has assessed the accuracy of this technique in infants, finding no association between diaphragm position and lung volume measured by inert gas washout.^7^ Using a larger sample size and different reference test,^12^ we have demonstrated similar results. There are several explanations for this finding. Fundamentally, a frontal chest radiograph is a 2-dimensional coronal image of the lungs, leaving the expansion of the lungs in the anterior and posterior direction unable to be examined.^8^ The more compliant and flexible infant thorax allows greater displacement along noncoronal planes compared with adults.^8,20^ Infants also perform more abdominal breathing and maintain a tonic diaphragm during respiration.^20,21^ This highlights that many factors contribute to the radiographic appearance of the lungs at any point in time beyond the level of respiratory support and phase of the respiratory cycle, especially during spontaneously breathing. Techniques that account for the 3-dimensional shape of the lungs using a series of chest radiographs have been developed and shown to accurately estimate lung volume in infants but are time consuming and not clinically practical.^22,23^

Our findings do not invalidate the use of chest radiographs in the NICU for other purposes, such as diagnosing pathologies.^10^ Chest radiographs provide additional information on abnormal aeration states such as radiolucency/opacity, bulging ribs, flattened diaphragm, or abnormal heart shape. Although we documented these features, due to the poor association with lung volume, we did not assess a potential composite score involving diaphragm position and other subjective measures of abnormal aeration status. Previous attempts have not significantly improved accuracy, likely due to the subjectivity of each.^7^ We contend that it is more appropriate to use a combination of clinical and diagnostic radiographic features to assess lung volume rather than diaphragm position alone. Oxygenation, carbon dioxide, and tidal volume all directly relate to lung volume in neonates, particularly significant hyperinflation or hypoinflation.^2,24,25^ Unlike chest radiographs, these measures allow for dynamic assessment after changes in mechanical support.^2,24,25^ In contrast, chest radiography is a static single snapshot of the lung and radiation exposure limits repeated use.

The ideal diaphragm position for infants is usually stated to be between the 7th and 9th posterior rib.^26^ In our study, despite a robust sample size, we found that 95% of diaphragm positions were distributed between the 8th and 10th posterior rib. Potentially many infants in our study had well-aerated lungs with optimized respiratory support as all infants were being closely monitored during the CT scan. Alternatively, most infants have a diaphragm position within normal limits regardless of the extent of respiratory pathology. Interestingly, the only other study^7^ to assess diaphragm position in neonates, and specifically those preterm, reported a range of 8 to 11 posterior ribs. In our study, 35% of the infants were intubated, and 61% had a degree of parenchymal opacification or consolidation. Thus, our study population was not standardized but had a diverse range of aeration states reflective of the NICU population. Furthermore, parenchymal opacification or consolidation was reported less on CREs than CT indicating that chest radiographs may lack the precision to detect more subtle parenchymal changes.^27^

We were surprised by the lack of a standardized definition on how to assess diaphragm position on chest radiograph. Although current guidelines suggest an optimal number of ribs to indicate lung inflation, there is minimal consensus in how to make this assessment, including assessing anterior or posterior ribs, the location of rib and diaphragm intersection (mediastinal, mid-clavicular, lateral intersection), and how best to categorize rib numbers (whole number vs rib space).^5,6,28^ Furthermore, the categorical nature and small range of potential rib number risks a large margin of difference if assessed inconsistently. As we demonstrated, diaphragm shapes relative to posterior rib level vary with potential interpretation and reproduction biases. Even using a standardized definition among neonatologists knowing they were being assessed, interobserver agreement was poor to moderate. Clinicians and radiologists may also have different priorities during assessment, with the radiologist focused more on the technical features of the study. As there is currently no single agreed-on definition to define diaphragm position on neonatal chest radiography, observer agreement is likely to be lower in clinical practice. This highlights the significant flaws of diaphragm position as a baseline accurate marker, regardless of its association with lung volume. None of the guidelines recommending the use of diaphragm position to inform respiratory support decisions comment on interpretation of right and left diaphragm discordance,^9,19,29^ an observation we found in 45% of CREs. In addition to creating confusion, this has important clinical implications. Targeting one side may result in overdistension or atelectasis of the other lung.

Lung volume should not be conflated with aeration states, such as atelectasis and overdistension, which are arguably more important than the absolute lung volume. The HU is the measure of tissue density on CT scan and is widely used to assess heterogeneity of lung aeration (SD HU).^30^ We demonstrated that the association between mean HU and SD HU with diaphragm position was even poorer than with lung volume. It is possible that this was due to the lack of standardization of the CREs to the inspiratory phase of the respiratory cycle. Although this may limit the generalizability of this study, it is reflective of clinical practice. Due to neonatal breathing patterns, chest radiographs are rarely able to be standardized to inspiration. In addition, there was diversity of respiratory support and parenchymal opacification or consolidation in the population suggesting that a variety of aeration states were present. Furthermore, many infants received intravenous contrast, which may skew the HU values and assessment of lung density. Regardless, in our study, the diaphragm position alone neither gave an accurate estimate of lung volume nor lung aeration.

The lack of precision of diaphragm position was due to the large range of lung volume at each posterior rib level (20-60 mL/kg). This is likely due to the fact that thoracic and diaphragm shape, rather than length alone, measured through midline diaphragm position, has a greater impact on lung volume.^31^ Normal lung volume in term infants is poorly understood, ranging from 18 mL/kg to 47 mL/kg.^1,32^ The ability of the semiautomated segmentation method to calculate lung volume in a large number of infants relatively easily provides a potential resource to determine normal lung volume ranges in infants. Lung ultrasound has been identified as a bedside, noninvasive tool that correlates well with global lung volumes from CT in adults with acute respiratory distress syndrome.^33^ Interestingly, a recent study^27^ in neonates reported a poor correlation between ultrasound calculated lung volume and rib number on chest radiographs. Electrical impedance tomography also correlates with global and regional lung volumes determined by CT and plethysmography, and neonatal systems are now commercially available.^34^ Although further research is required, both lung ultrasound and electrical impedance tomography appear to identify atelectasis and recruitment similarly to oxygenation.^27,34,35^ As we have shown in this study, reliance on a singular imaging tool maybe misleading. We suggest that bedside assessment of lung inflation and respiratory status should first be by clinical measures, and imaging then can be used during or after optimization.

The semiautomated segmentation method may have underestimated the total lung volume. CT is an accepted accurate measure of lung volume in adult and pediatric ICUs.^36,37^ As part of this study, we demonstrated a high intraparticipant and interparticipant agreement using the segmentation tool to calculate aerated lung (eMethods in Supplement 1). Semiautomated segmentation may not accurately represent total lung volume when there is significant parenchymal opacification or consolidation. Complete manual segmentation has been reported,^38^ but it is too time consuming to be practical in a large population. Despite this, the association between radiographic diaphragm position and lung volume using only CT scans without parenchymal opacification or consolidation (as a marker of fully aerated lungs) was similarly weak.

### Strengths and Limitations

Our study has some strengths not already discussed. The range and variety in our study population and technical elements, such as image rotation, reflect the difficulties in measuring lung volume in infants within a research setting and has arguably contributed to the lack of data to support chest radiography to guide respiratory support. Thus, the use of CT paired with the chest scout topogram is a strength, allowing a large sample size. However, a topogram differs from a chest radiograph. Despite the technical differences, the position of the diaphragm relative to posterior ribs is unlikely to be clinically different, especially in the small neonatal chest.

Our study also has limitations. As a retrospective observational study, we have been unable to assess the dynamic effect of changing lung volume on the posterior rib level of the diaphragm position within an infant. Although a prospective validation study would be ideal, this would raise safety and ethical concerns related to radiation exposure and potential harmful lung volume states. The masking and randomization of infants within the study design negates some of the potential biases of a retrospective cohort. The need for a retrospective design limited CT scans to those primarily ordered for cardiac reasons and in term infants. Although preterm infants are the most common population in the NICU, the greater compliance of the preterm chest wall is likely to reduce the reliability of diaphragm position. We also excluded infants with cardiac diagnoses that may result in an unrepresentative contribution of the heart to total thoracic volume. Finally, although the CRE and CT images did not occur during the same inflation, it allowed as near a temporal comparison as is practical.

## Conclusions

Results of this cross-sectional study suggest that despite long-standing clinical acceptance, the diaphragm position as measured through the number of posterior ribs on a chest radiograph may not be sufficiently accurate for use in practice as a surrogate of lung volume. We would caution against use to guide respiratory support decisions. Until alternative noninvasive methods of assessing lung volume are available, we recommend guidelines use clinical measures.

## Notes

Supplement 1.
eMethods.
**eFigure 1.** Representative Images to Demonstrate the Method of Defining Diaphragm Position Based Upon Posterior Rib Number **eFigure 2.** Chest Topogram (Chest Radiograph Equivalent) Identifying the Apex-Diaphragm Distance (mm) **eFigure 3.** Syngo.via CT Axial, Coronal, Sagittal, and VRT Images After Application of Region Growing Method and Fine-Tuning Tools **eFigure 4.** Scatterplot Demonstrating Interobserver Agreement Between 3 Observers' Calculation of V_L_ From CT Images **eFigure 5.** Bland-Altman Plot Demonstrating Interobserver Agreement Between 3 Observers of Calculation of V_L_ From CT Images **eFigure 6.** Scatterplot Demonstrating Intraobserver Agreement Between 2 Observers of Calculation of V_L_ From CT Images **eFigure 7.** Scatterplot Demonstrating the Interobserver Agreement Between 3 Investigators for Calculation of Diaphragm Position on Chest Radiograph Equivalent (Topogram) **eFigure 8.** Scatterplot Demonstrating the Interobserver Agreement Between 3 Investigators for Chest Radiograph Equivalent (Topogram) Apex-Diaphragm Distance (mm) **eFigure 9.** STROBE Diagram **eTable.** Additional Subject Characteristics (Supplement to Manuscript Table 1) **eFigure 10.** Relationship Between Total Lung Volume and Diaphragm Position Defined by the Smaller Posterior Rib **eFigure 11.** Absolute Relationships Between Total Lung Volume and Diaphragm Position **eFigure 12.** Relationship Between Total Lung Volume and Diaphragm Position in Chest Radiograph Equivalent (CRE) Identified as Overdistended and Nonoverdistended CREs **eFigure 13.** Relationship Between Total Lung Volume and Diaphragm Position in Chest Radiograph Equivalent (CRE) Identified as Atelectatic and Nonatelectatic CREs **eFigure 14.** Relationship Between Hounsfield Units and Diaphragm Position for the HU_mean_ and HU_SD_ **eFigure 15.** Additional HU Data **eFigure 16.** Total, Left, and Right V_L_ Compared With Respective Apex-Diaphragm Distance

Supplement 2.
Data Sharing Statement.
