# Supplementary material for: Diaphragm Position on Chest Radiograph to Estimate Lung Volume in Neonates
Source: JAMA Pediatr. 2025 Jul 21;179(9):1034–42. doi: 10.1001/jamapediatrics.2025.2108 (PMC12281396; doi:10.1001/jamapediatrics.2025.2108)
Supplement: Supplement 1. — eMethods. eFigure 1. Representative Images to Demonstrate the Method of Defining Diaphragm Position Based Upon Posterior Rib Number eFigure 2. Chest Topogram (Chest Radiograph Equivalent) Identifying the Apex-Diaphragm Distance (mm) eFigure 3. Syngo.via CT Axial, Coronal, Sagittal, and VRT Images After Application of Region Growing Method and Fine-Tuning Tools eFigure 4. Scatterplot Demonstrating Interobserver Agreement Between 3 Observers' Calculation of VL From CT Images eFigure 5. Bland-Altman Plot Demonstrating Interobserver Agreement Between 3 Observers of Calculation of VL From CT Images eFigure 6. Scatterplot Demonstrating Intraobserver Agreement Between 2 Observers of Calculation of VL From CT Images eFigure 7. Scatterplot Demonstrating the Interobserver Agreement Between 3 Investigators for Calculation of Diaphragm Position on Chest Radiograph Equivalent (Topogram) eFigure 8. Scatterplot Demonstrating the Interobserver Agreement Between 3 Investigators for Chest Radiograph Equivalent (Topogram) Apex-Diaphragm Distance (mm) eFigure 9. STROBE Diagram eTable. Additional Subject Characteristics (Supplement to Manuscript Table 1) eFigure 10. Relationship Between Total Lung Volume and Diaphragm Position Defined by the Smaller Posterior Rib eFigure 11. Absolute Relationships Between Total Lung Volume and Diaphragm Position eFigure 12. Relationship Between Total Lung Volume and Diaphragm Position in Chest Radiograph Equivalent (CRE) Identified as Overdistended and Nonoverdistended CREs eFigure 13. Relationship Between Total Lung Volume and Diaphragm Position in Chest Radiograph Equivalent (CRE) Identified as Atelectatic and Nonatelectatic CREs eFigure 14. Relationship Between Hounsfield Units and Diaphragm Position for the HUmean and HUSD eFigure 15. Additional HU Data eFigure 16. Total, Left, and Right VL Compared With Respective Apex-Diaphragm Distance [file jamapediatr-e252108-s001.pdf]

Supplementary Online Content

Dahm SI, Sett A, Gunn EF, et al. Diaphragm position on chest radiograph to estimate lung volumes in neonates. *JAMA Pediatr*. Published online July 21, 2025. doi:10.1001/jamapediatrics.2025.2108

eMethods.

eFigure 1. Representative Images to Demonstrate the Method of Defining Diaphragm Position Based Upon Posterior Rib Number

eFigure 2. Chest Topogram (Chest Radiograph Equivalent) Identifying the Apex-Diaphragm Distance (mm)

eFigure 3. Syngo.via CT Axial, Coronal, Sagittal, and VRT Images After Application of Region Growing Method and Fine-Tuning Tools

eFigure 4. Scatterplot Demonstrating Interobserver Agreement Between 3 Observers' Calculation of V_L From CT Images

eFigure 5. Bland-Altman Plot Demonstrating Interobserver Agreement Between 3 Observers of Calculation of V_L From CT Images

eFigure 6. Scatterplot Demonstrating Intraobserver Agreement Between 2 Observers of Calculation of V_L From CT Images

eFigure 7. Scatterplot Demonstrating the Interobserver Agreement Between 3 Investigators for Calculation of Diaphragm Position on Chest Radiograph Equivalent (Topogram)

eFigure 8. Scatterplot Demonstrating the Interobserver Agreement Between 3 Investigators for Chest Radiograph Equivalent (Topogram) Apex-Diaphragm Distance (mm)

eResults.

eFigure 9. STROBE Diagram

eTable. Additional Subject Characteristics (Supplement to Manuscript Table 1)

eFigure 10. Relationship Between Total Lung Volume and Diaphragm Position Defined by the Smaller Posterior Rib

eFigure 11. Absolute Relationships Between Total Lung Volume and Diaphragm Position

eFigure 12. Relationship Between Total Lung Volume and Diaphragm Position in Chest Radiograph Equivalent (CRE) Identified as Overdistended and Nonoverdistended CREs

eFigure 13. Relationship Between Total Lung Volume and Diaphragm Position in Chest Radiograph Equivalent (CRE) Identified as Atelectatic and Nonatelectatic CREs

© 2025 Dahm SI et al. *JAMA Pediatrics*.

eFigure 14. Relationship Between Hounsfield Units and Diaphragm Position for the HU_{mean} and HU_{SD}

eFigure 15. Additional HU Data

eFigure 16. Total, Left, and Right V_L Compared With Respective Apex-Diaphragm Distance

This supplementary material has been provided by the authors to give readers additional information about their work.

© 2025 Dahm SI et al. *JAMA Pediatrics*.

eMETHODS

a) Methods for defining diaphragm position relative to posterior rib number

There is no universally agreed method of defining diaphragm position using the posterior ribs.[1,2,3] For this study the diaphragm position was defined as the last posterior rib that completely intersects the diaphragm or had the diaphragm positioned entirely within the rib (eFigure 1). The rib above was used if no posterior rib intersected the diaphragm. When the diaphragm involved two ribs, but did not completely transect the lower rib, the upper rib was used if <50% of the intercostal space between ribs included lung fields and vice versa. This approach was developed under consultation with two experienced Radiologists (PR and RG). All assessors were trained by RG and 'calibrated' against RG assessment in a series of test images. To do so, 30 CXRs were randomly selected for assessment by all neonatologists and one radiologist (RG) to determine reproducibility and reliability of the diaphragm position using the applied definition.

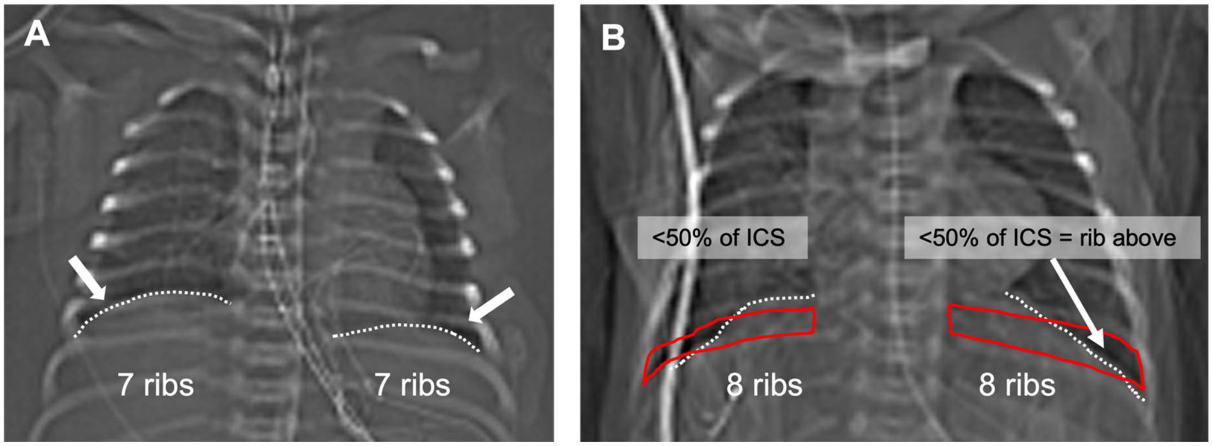

eFigure 1. Representative images to demonstrate the method of defining diaphragm position based upon posterior rib number. **A.** The diaphragm position was defined as the last posterior rib (arrows) that completely intersected the diaphragm (dotted lines) or completely contained the diaphragm (right diaphragm). If the diaphragm did not intersect any rib the rib above was used (left diaphragm). **B.** When the diaphragm involved two ribs the upper rib was used if <50% of the intercostal space (ICS; outlined in red) includes lung field and vice versa.

© 2025 Dahm SI et al. *JAMA Pediatrics*.

b) Methods for defining vertical apex-diaphragm distance

The left and right lung vertical apex-diaphragm distance (mm) was also calculated (eFigure 2).

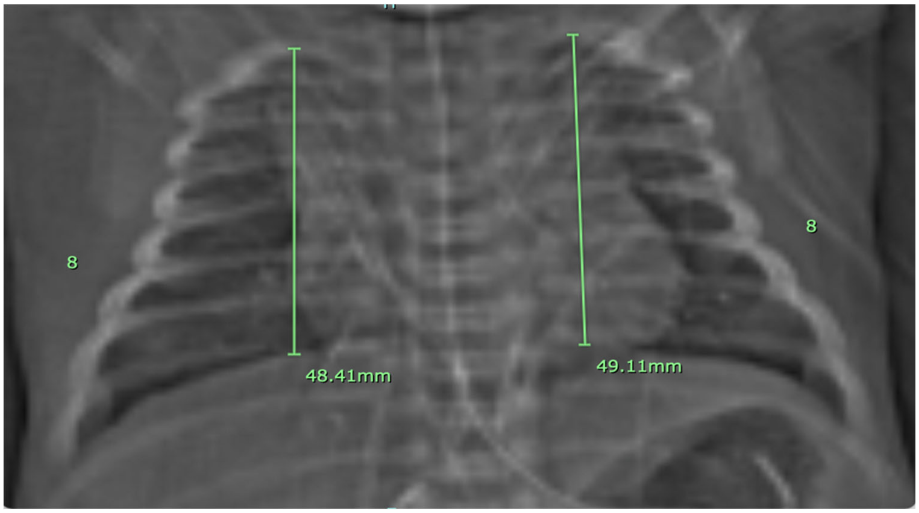

eFigure 2. Chest topogram (chest radiograph equivalent) identifying the apex-diaphragm distance (mm). The apex-diaphragm distance is measured from the apex of the lung as defined by the top of the first rib, to the diaphragm directly below in a straight line.

c) Semi-automated tissue segmentation to determine lung volume and other CT-measures of aeration

Lung volume (mL) was calculated from included CT scans using the Siemens syngio.via (Simens Healthcare GmbH, Erlangen, Germany) semi-automated tissue segmentation tool. Each scan was assigned a Multi-Modal Reading workflow prior to loading the scan. The region growing tool (single click mode and airway setting) was used in the Anatomy Visualiser program. Aerated lung tissue was then 'marked' from the resultant image manually through subjective assessment.

Aerated lung boundaries were determined through subjective assessment by each observer. Marked regions were refined using the 'Fill Holes' and 'Expand' tools. The selected lung areas underwent quantification though the Syngo.via application, which computed various metrics, including aerated lung volume (V_L), maximum HU, minimum HU, mean HU and standard deviation (SD) of the HU. This was completed firstly for the left lung, then repeat for the right lung and the summative measurement was achieved through amalgamation of the individual lung segments, facilitated by use of the Venn diagram tool. Results were presented for all three segmented lung fields; Left lung, Right lung and Total lung (eFigure 3). A snapshot of the computation was captured and exported to the hospital Picture Archiving and Communication *System (PACS)* for subsequent evaluation.

© 2025 Dahm SI et al. *JAMA Pediatrics*.

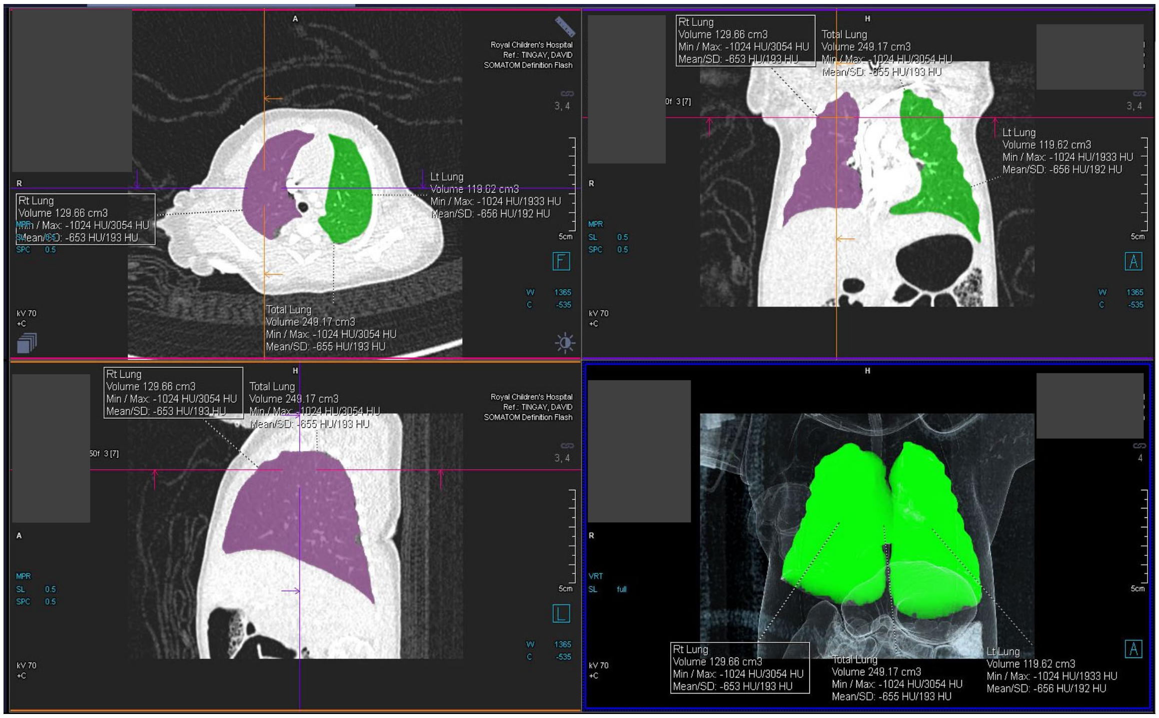

eFigure 3. Syngo.via CT axial, coronal, sagittal and VRT images after application of Region Growing method and fine-tuning tools. The purple region indicates the right lung volume analysis. The green region (top left and right) indicates the left lung volume analysis. The green region (bottom right) indicates the 3D representation of total aerated V_L.

© 2025 Dahm SI et al. *JAMA Pediatrics*.

Areas of consolidation that could not easily be differentiated from the surrounding structures were not included in the automated segmentation. Each observer was asked to independently comment on the presence of atelectasis and quantify as mild, moderate, or severe based on both the amount and density of atelectatic tissue. Atelectasis was defined by the characteristic morphology of tissue opacities. Additionally, CT scans that did not include all lung tissue at time of scanning were identified and reviewed by two investigators to determine their inclusion or exclusion depending on percentage of lung not included in the scan. Scans were excluded if there was missing lung tissue in 2 or more planes of the CT scan. All assessors performed the segmentation blinded to the clinical details of the neonate and results of the other assessors.

d) Inter-observer and intra-observer agreement of CT semi-automated segmentation tool

The inter-observer and intra-observer agreement of the semi-automated CT segmentation tool was determined from the CT scans of the first 30 eligible infants, and analysis completed prior to analysis of the remaining study population. The analysis was conducted as a separate university project by EG.

All 30 CT scans were assessed by three independent observers (FR, SD, EG), chosen as each had differing levels of radiological and clinical experience; radiographer specialising in CT (FR), paediatric trainee medical office (SD) and medical student (EG). Lung tissue was defined using the semi-automated segmentation tool as detailed above, and V_L and HU determined for the whole lung and right and left lungs from each of the 30 CT scans by all three observers (inter-observer agreement). Each CT image was also assessed twice by SD and EG approximately three weeks apart to determine the intra-observer agreement. For intra-observer agreement the CT images were allocated different ID numbers at each assessment to minimise recall bias.

Inter- and intra-observer agreement was assessed using an Intraclass Correlation Coefficient (ICC) with two-way random effects model and Bland-Altman analysis. A minimum clinically acceptable limit of agreement of >0.8 was used. Significance was set at $p<0.05$. Analysis was performed using R (R: A language and environment for statistical computing. R Foundation for Statistical Computing, Vienna, Austria, 2022).

© 2025 Dahm SI et al. *JAMA Pediatrics*.

Inter-observer agreement

Inter-observer agreement was excellent between SD (observer 1) and EG (observer 2) with an ICC of 0.98 (95% CI 0.95-0.99), and good between EG (observer 2) and FR (observer 3) with an ICC of 0.86 (95% CI 0.73-0.93) (eFigure 4).

The Bland-Altman plot between observer 1 (SD) and observer 2 (EG) demonstrated a small and clinically insignificant negative bias of -1.2 (95% limit of agreement (LOA) -23.1 to 20.7) mL/kg (eFigure 5A). The bias between observer 2 (EG) and observer 3 (FR) was also clinically insignificant but positive; 3.5 (-68.1 to 75.2) mL/kg (eFigure 5B).

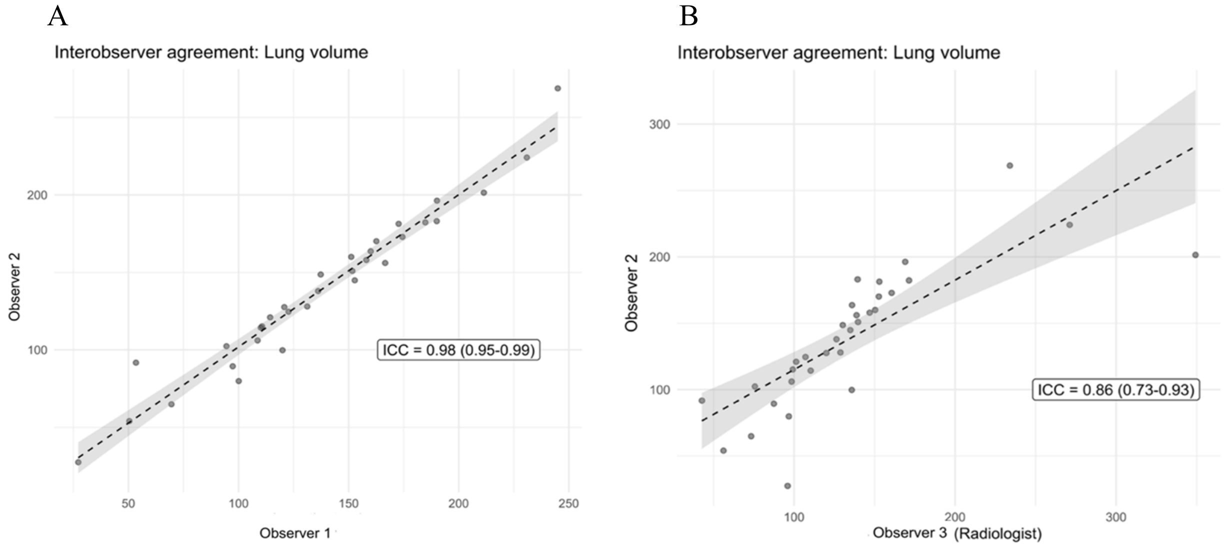

eFigure 4. Scatterplot demonstrating the inter-observer agreement between three observers (**A**; Observer 1 and 2, **B**; Observer 2 and 3) calculation of V_L from the CT images using the semi-automated segmentation tool. Black dots represent individual infants, and the dotted black line represents the line of best fit, with the grey shaded area representing the 95% confidence interval. Intraclass Correlation Coefficient analysis (ICC) shown in each panel.

A

B

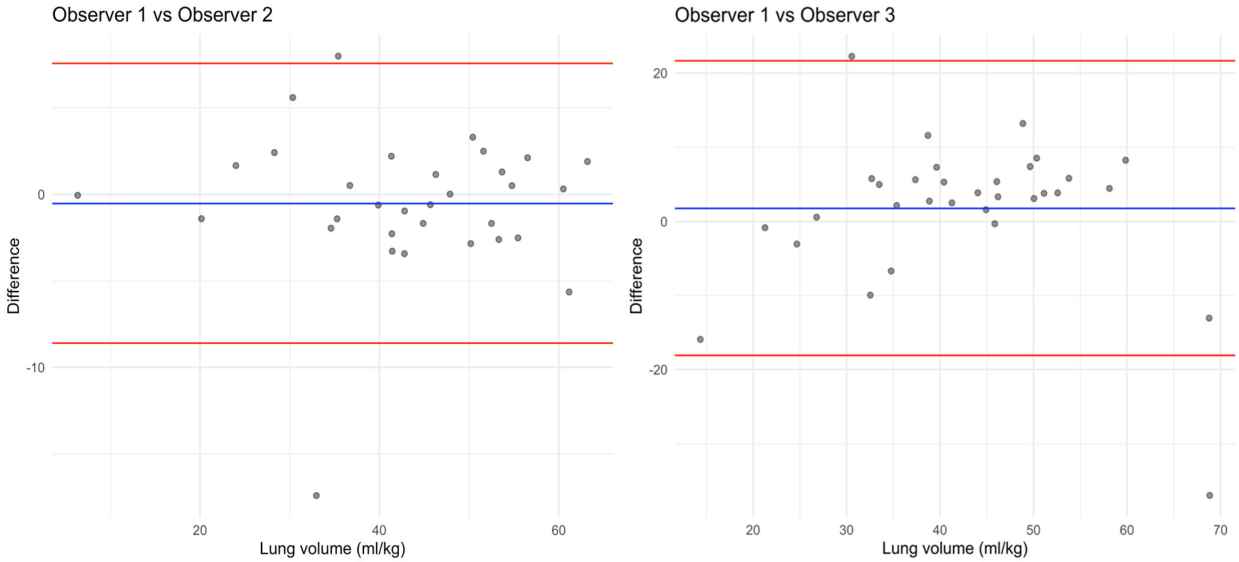

eFigure 5. Bland-Altman plot demonstrating the inter-observer agreement between three observers' calculation of V_L from the CT images using the semi-automated segmentation tool. The blue line indicates the mean difference (bias), and the red lines indicate the 95% limit of agreement. Blue dots represents an individual infant. Observer 1 and 2 showed a bias of -1.2 (95% LOA of -23.1 to 20.7) mL/kg, regression coefficient -0.0069 (**A**), Observer 1 and Observer 3 showed a bias of 3.5 (95% LOA of -68.1 to 75.2) mL/kg and regression coefficient of -0.18 (**B**).

© 2025 Dahm SI et al. *JAMA Pediatrics*.

Intra-observer agreement

The ICC between data set one and data set two for Observer 1 (SD) was 0.97 (95% CI 0.91-0.99) and the ICC for Observer 2 (EG) was 0.99 (95% CI 0.97-0.99) (eFigure 6).

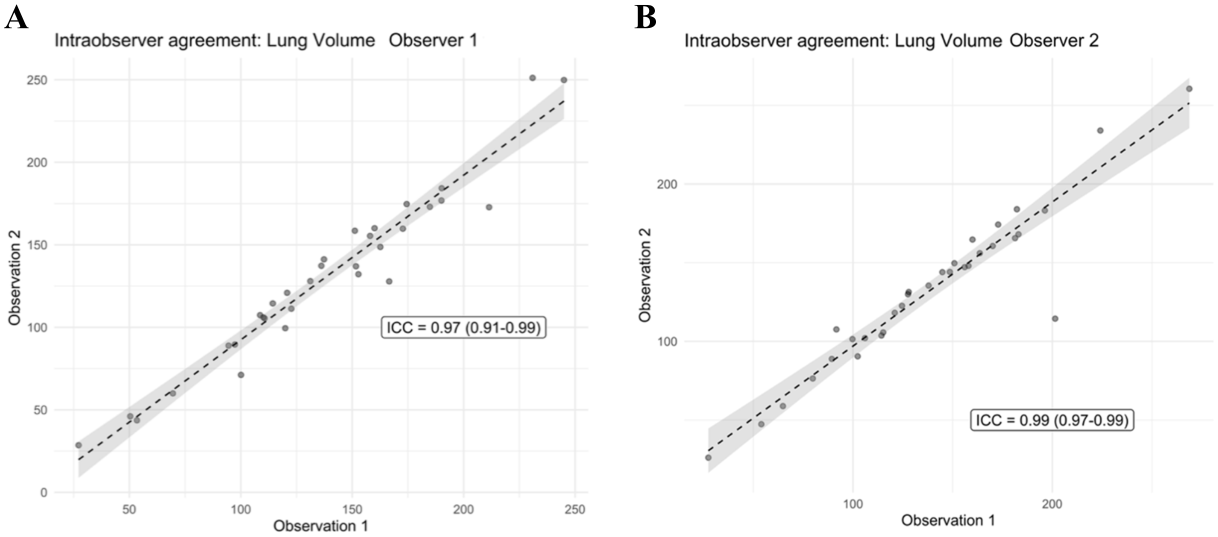

eFigure 6. Scatterplot demonstrating the intra-observer agreement between the calculation of V_L from the CT images using the semi-automated segmentation tool from the same images at two separate occasions by two observers (**A**; SD, **B**; EG). Black dots represent individual infants, and the dotted black line represents the line of best fit, with the grey shaded area representing the 95% confidence interval. Intraclass Correlation Coefficient (ICC) shown in each panel.

© 2025 Dahm SI et al. *JAMA Pediatrics*.

e) Inter-observer agreement of determination of diaphragm position from chest topogram

The same first 30 infants used to determine the inter- and intra-observer agreement of the CT semi-automated segmentation tool were analysed for inter-agreement of the method of determining the diaphragm position from chest topogram. The apex-diaphragm distance was also determined. All 30 chest topograms were assessed by three independent observers (AS, DT, DS). These observers were chosen as each are practicing neonatologists and thus representative of clinicians who may use rib position on CXR in the NICU.

Inter-observer agreement of the categorical variable of rib number was assessed using a Cohens Kappa Coefficient (k). Inter-observer agreement of the apex-diaphragm distance was assessed using an Intraclass Correlation Coefficient (ICC) with two-way random effects model. A minimum clinically acceptable limit of agreement of >0.8 was used. Significance was set at $p<0.05$. Analysis was performed using R (R: A language and environment for statistical computing. R Foundation for Statistical Computing, Vienna, Austria, 2022).

© 2025 Dahm SI et al. *JAMA Pediatrics*.

Inter-observer agreement

eFigure 7 shows the relationship between diaphragm position for all 3 observers for the right and left hemithorax. The agreement between observers was only poor-moderate (0.20-0.67). There was good agreement between observers for the left and right hemithorax apex-diaphragm distance (eFigure 8).

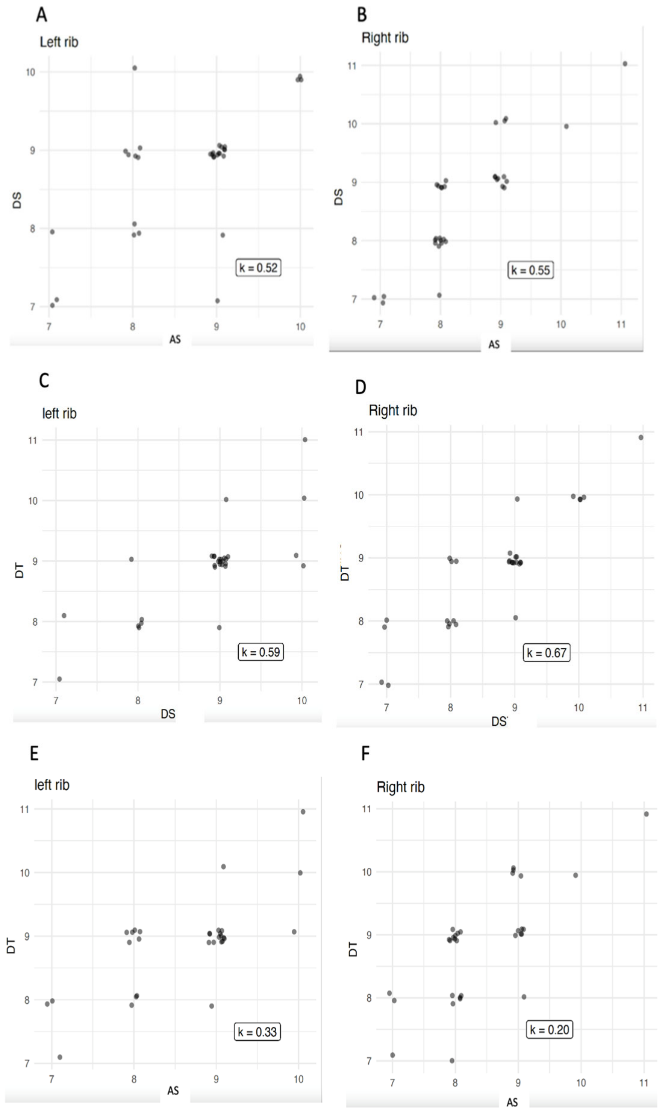

eFigure 7. Scatterplot demonstrating the inter-observer agreement between three investigators (AS, DS, DT) for calculation of diaphragm position on chest radiograph equivalent (topogram) based upon posterior rib number in the left and right hemithoraces. Each black dot represents an individual data point. Cohens Kappa coefficient (k) for each permutation shown in respective panel.

© 2025 Dahm SI et al. *JAMA Pediatrics*.

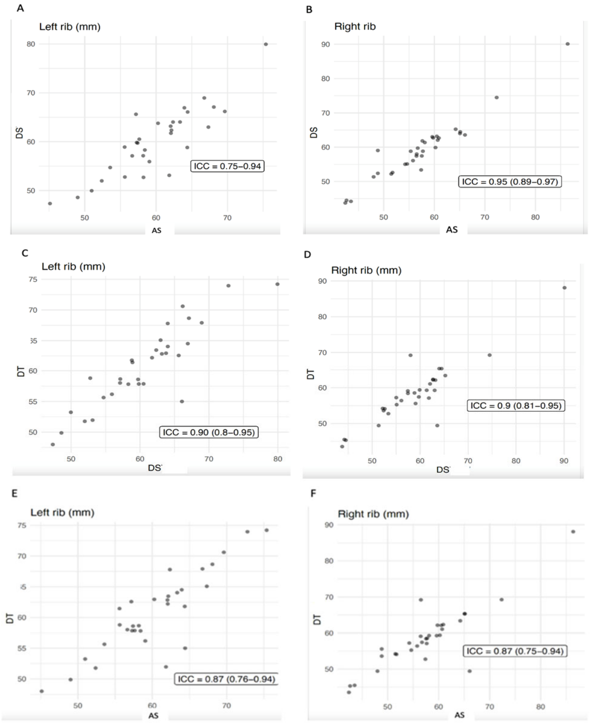

eFigure 8. Scatterplot demonstrating the inter-observer agreement between three investigators (AS, DS, DT) for chest topogram apex-diaphragm distance (mm) assessment for the left (left hand panels) and right (right hand panels) hemithoraces. Each black dot represents an individual data point. Intraclass Correlation Coefficient (ICC) and 95% CI of each permutation shown in respective panel.

© 2025 Dahm SI et al. *JAMA Pediatrics*.

f) Sample size calculation and Statistical Analysis

Statistical advice was sought from the Clinical Epidemiology and Biostatistics Unit (CEBU) (Prof Susan Donath) at the Murdoch Children's Research Institute, Melbourne, Australia at the commencement of this study (prior to prospective study registration), then at conclusion of data collection (prior to data analysis) and finally after data analysis. It was anticipated that the rib number values would range from 6-11 ribs, however with an uneven distribution of this categorical variable, with data favouring 8-10 ribs. As such, a feasibility sample size of at least 200 CT scans was determined to provide a robust number of subjects for each potential rib value. Displaying data represented a challenge as the data of this study is being presented as categorical/ordinal (rib number) compared with continuous (V_L). Presenting the relationship between V_L and rib number as a box and whisker plot was determined to be the most clinically useful representation, with strength of association between variables calculated using Kendal Tau's correlation coefficient and linear regression analysis.

References

1. Clark RH, Gerstmann DR, Null DM, Jr., de Lemos RA. Prospective randomized comparison of high-frequency oscillatory and conventional ventilation in respiratory distress syndrome. Pediatrics 1992; 89(1): 5-12.
2. Yasmeen T. AA. High Frequency Oscillatory Ventilation (HFOV): A guide to the use of HFOV in the neonate. 2020. https://www.clinicalguidelines.scot.nhs.uk/nhsggc-guidelines/nhsggc-guidelines/neonatology/high-frequency-oscillatory-ventilation-hfov-a-guide-to-the-use-of-hfov-in-the-neonate/#S25.
3. Castro D, Naqvi A, Manson D, Flavin M, Vandenkerkhof E, Soboleski D. Novel Method to Improve Radiologist Agreement in Interpretation of Serial Chest Radiographs in the ICU. Journal of Clinical Imaging Science 2015; 5(1).
4. Mansoor A, Cerrolaza JJ, Perez G, Biggs E, Okada K, Nino G, Linguraru MG. A Generic Approach to Lung Field Segmentation From Chest Radiographs Using Deep Space and Shape Learning. *IEEE Trans Biomed Eng* 2020; 67: 1206-1220.

© 2025 Dahm SI et al. *JAMA Pediatrics*.

eResults.

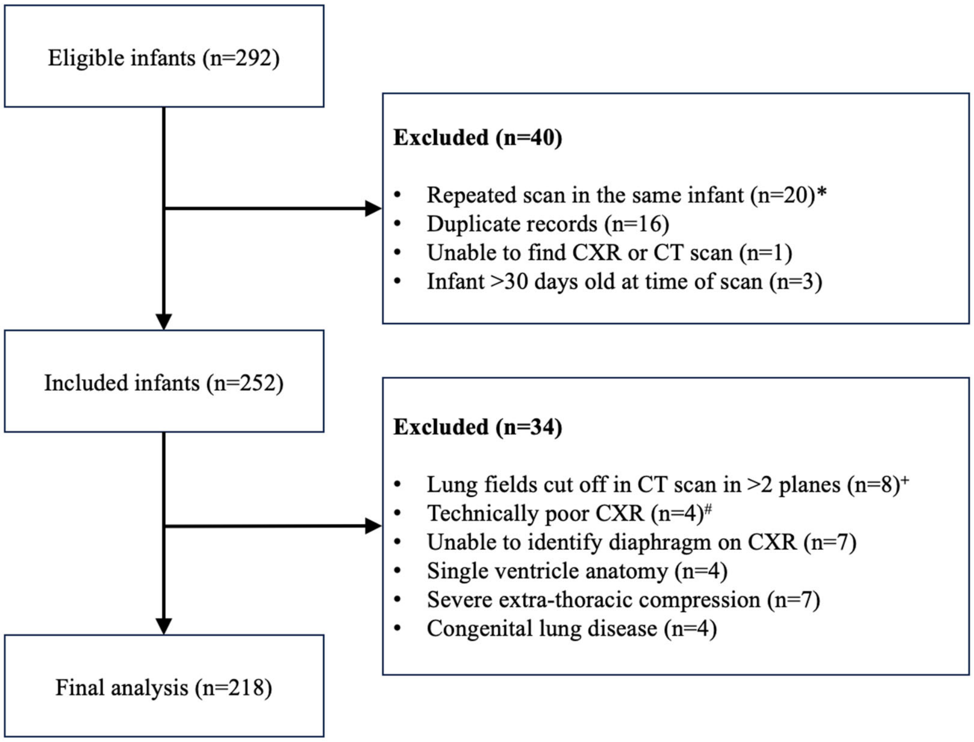

eFigure 9. STROBE diagram.
*First scan analysed and subsequent scans excluded from analysis. [+]Decision to exclude an infant based on CT scans where the lung fields were cut off after separate assessment and agreement by two investigators. [#]Defined as thorax and/or lung fields deemed grossly distorted due to rotation or poor exposure (n=3), or CXR did not include all lung fields (n=1).

© 2025 Dahm SI et al. *JAMA Pediatrics*.

eTable. Additional subject characteristics

Primary Diagnosis: Details of the 11 infants classified as 'other' in manuscript Table 1.<br>Pulmonary hypertension 3 (1.5%); Tracheomalacia 2 (1%); Vascular ring 2 (1%); Embolism 2 (1%); Neck mass 1 (0.5%); Mediastinal mass 1 (0.5%).
Comorbidities: Details of the 50 infants classified as 'other' in manuscript Table 1.<br>Dextrocardia 5 (2%); Known genetic condition 12 (5%); extra-thoracic disease 16)7%); extra-pulmonary disease 14 (6%); Intrinsic pulmonary disease 3 (1.5%).
Indication for CT: Details of the 11 infants classified as 'other' in manuscript Table 1.<br>Failed extubation 2 (1%); extra-thoracic reason 9 (4%).

© 2025 Dahm SI et al. *JAMA Pediatrics*.

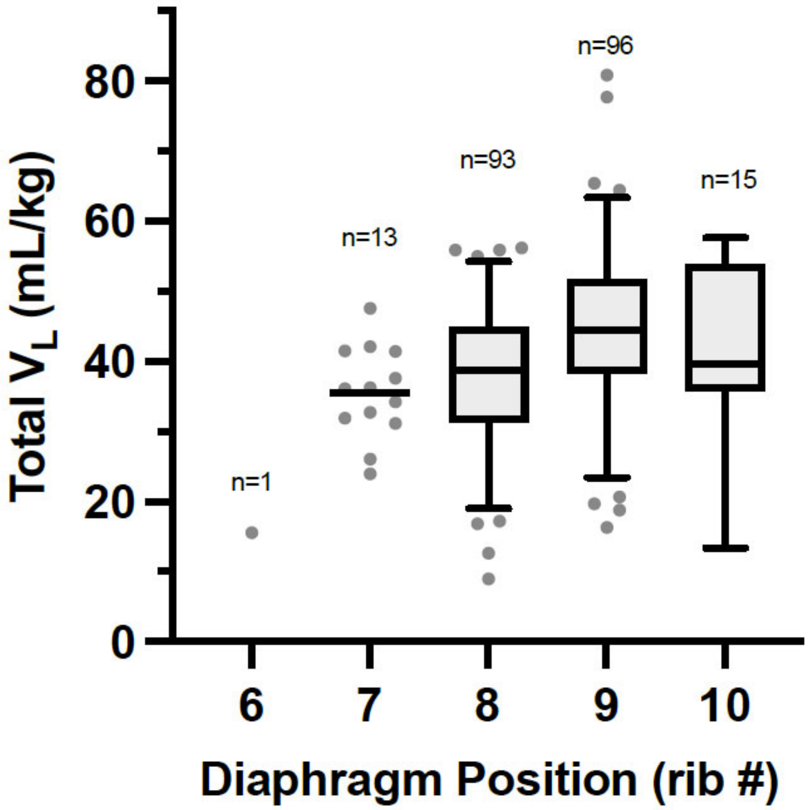

eFigure 10. Relationship between total lung volume (V_L; mL/kg) and diaphragm position defined by the smaller posterior rib (rib #). Where left and right diaphragm position differed, the smaller rib number was selected for comparison. Boxes interquartile range and median (horizontal line). Whiskers represent 1.5* interquartile range. Individual data points shown as grey circles. Number of infants for each diaphragm position shown in Figure. Due to small numbers at 6 and 7 ribs all data points (and median value rib #7) shown.

© 2025 Dahm SI et al. *JAMA Pediatrics*.

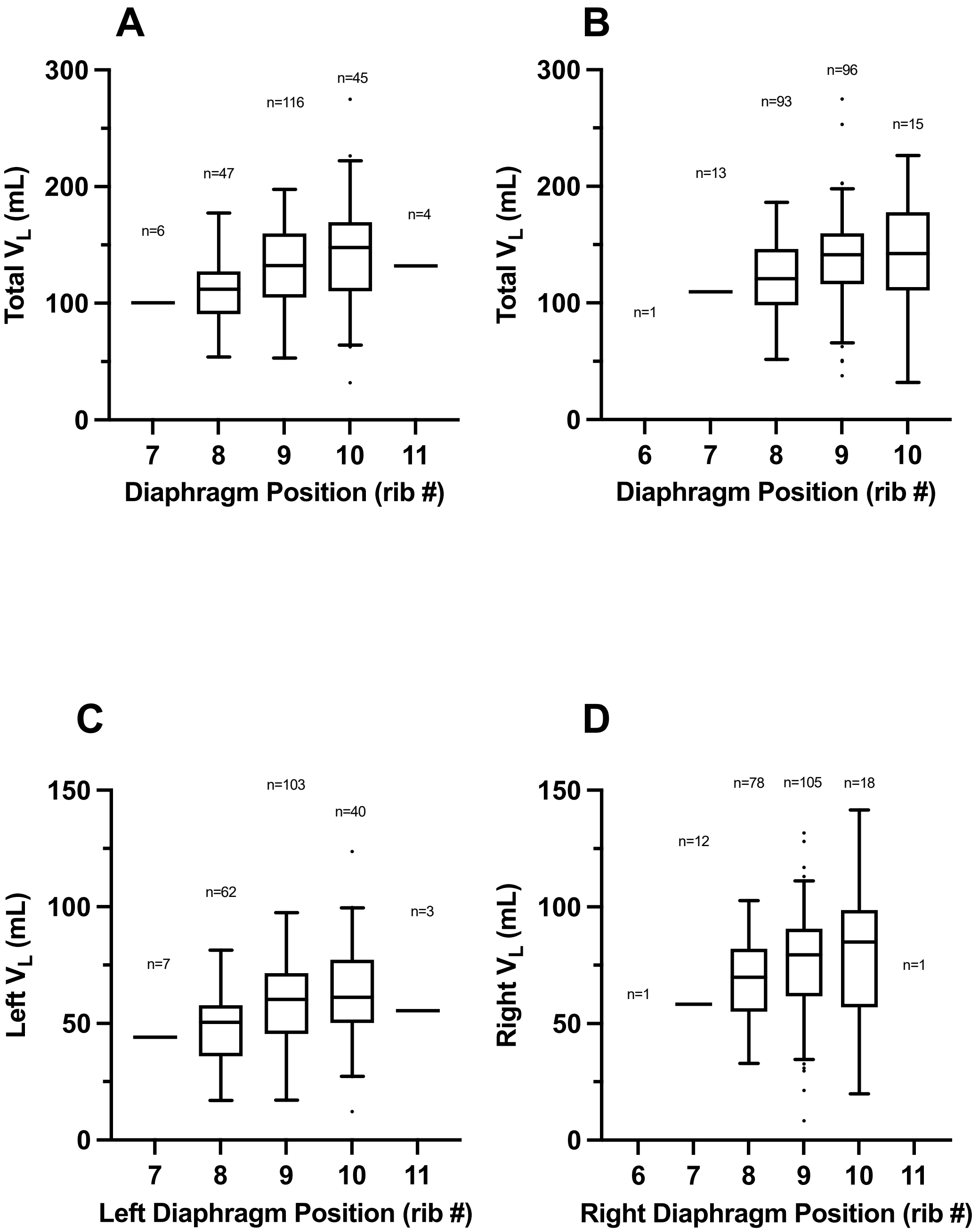

eFigure 11. Absolute relationships between total lung volume (V_L; mL) and diaphragm position (rib #) for greatest rib number (A), lowest rib number (B), left (C) and right (D). Symbols and descriptors as per eFigure 10. Kendal Tau's correlation coefficient (95% CI); (**A**) τ = 0.21 (0.12,0.30), (**B**) τ = 0.22 (0.12,0.32), (**C**) τ = 0.23 (0.23,0.32), (**D**) τ = 0.18 (0.08,0.28).

© 2025 Dahm SI et al. *JAMA Pediatrics*.

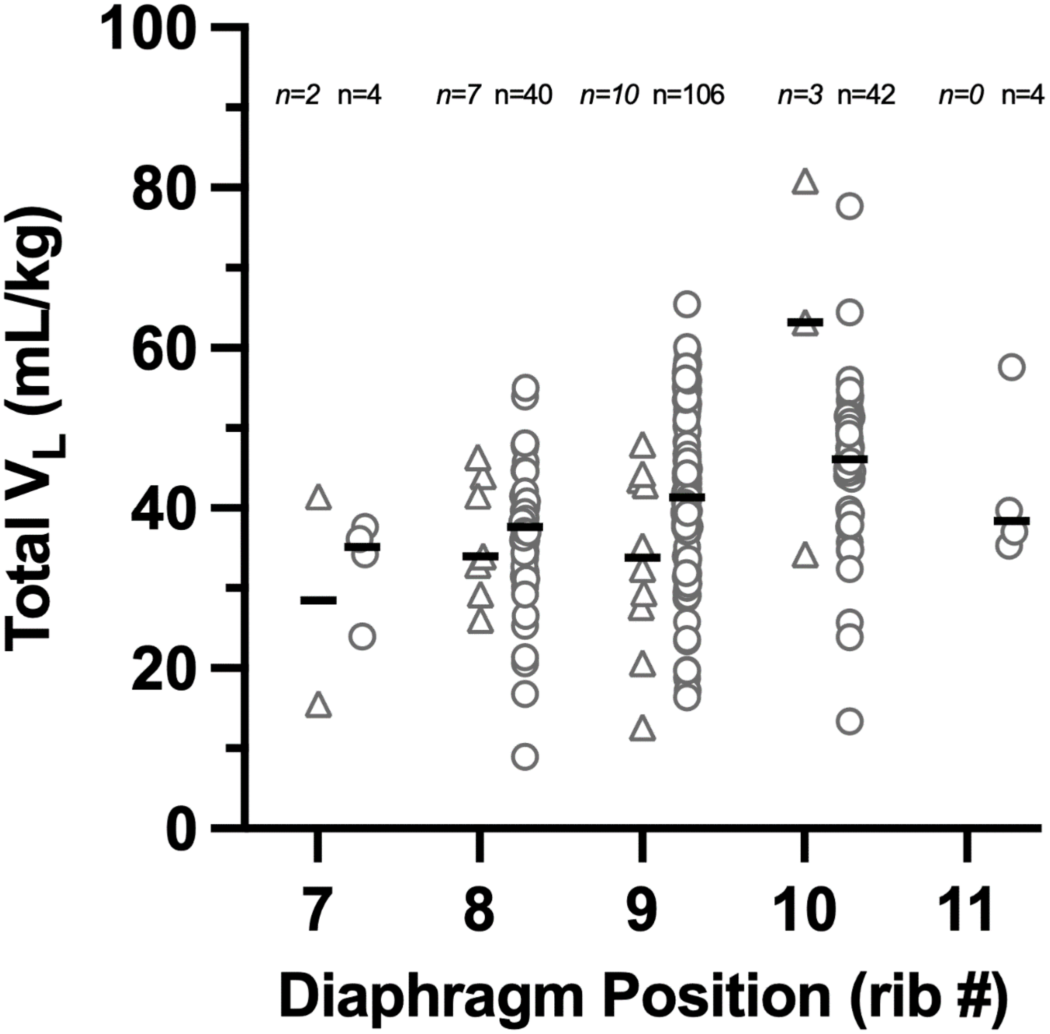

eFigure 12: Relationship between total lung volume (V_L; mL/kg) and diaphragm position in chest radiograph equivalent (CRE) identified as overdistended (grey triangles, italics text) and non-overdistended CREs (grey circles, non-italic text). Where left and right diaphragm position differed, the smaller rib number was selected for comparison. Scatterplot with all data points shown. Lines indicates median. Number of infants for each diaphragm position shown in Figure. Due to small samples sizes, no statistical analysis performed.

© 2025 Dahm SI et al. *JAMA Pediatrics*.

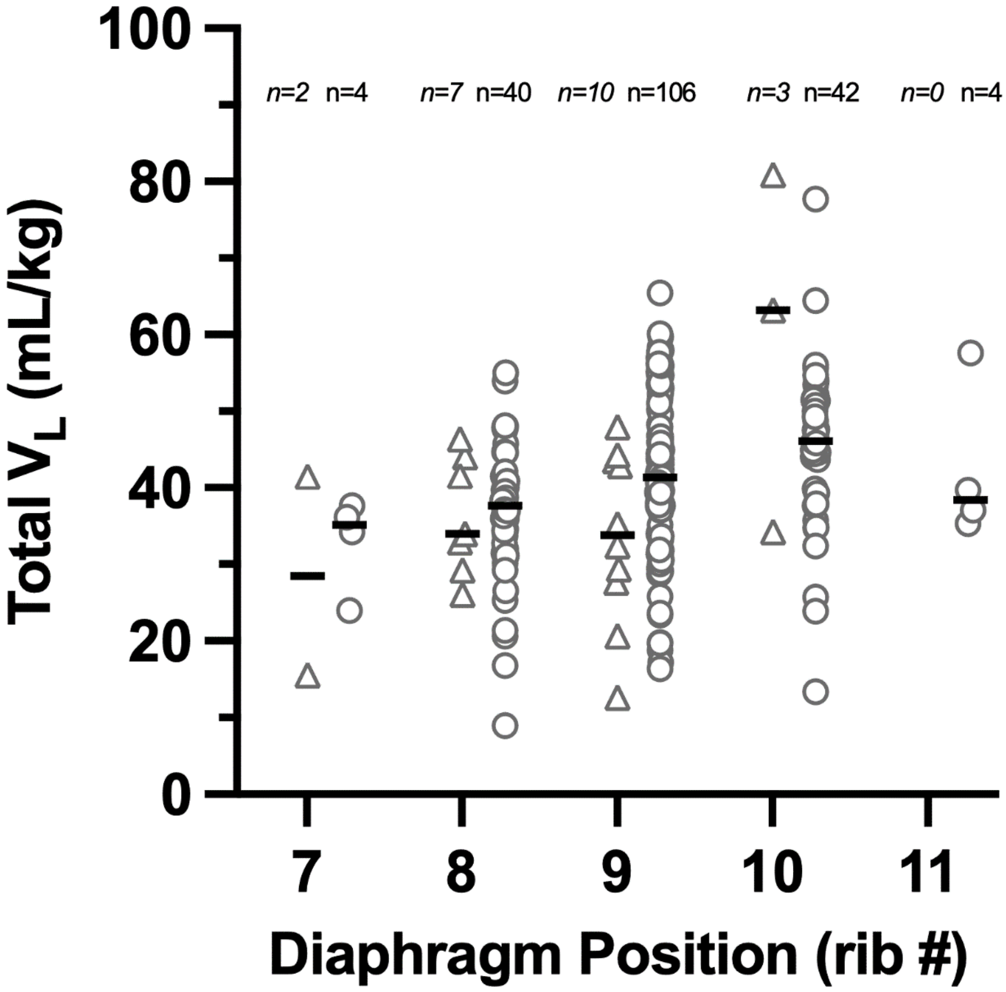

eFigure 13. Relationship between total lung volume (V_L; mL/kg) and diaphragm position in chest radiograph equivalent (CRE) identified as atelectatic (grey triangles, italics text) and non-atelectatic CREs (grey circles, non-italic text). Where left and right diaphragm position differed, the smaller rib number was selected for comparison. Scatterplot with all data points shown. Lines indicates median value. Number of infants for each diaphragm position shown in Figure. Due to small samples sizes, no statistical analysis performed.

© 2025 Dahm SI et al. *JAMA Pediatrics*.

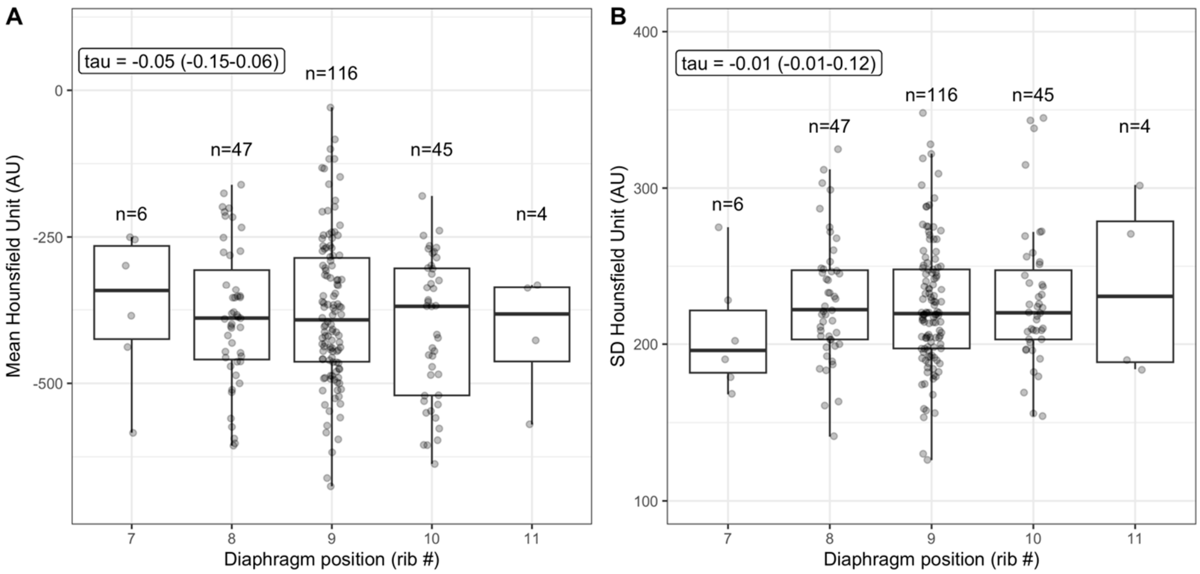

eFigure 14. Relationship between HU and diaphragm position (rib #) for the HU_{mean} (A) and HU_{SD} (B). Where left and right diaphragm position differed, the greater rib number was selected for comparison. Symbols and descriptors as per eFigure 10. Normal HU range for lung tissue (-440, -700 HU).[4]

© 2025 Dahm SI et al. *JAMA Pediatrics*.

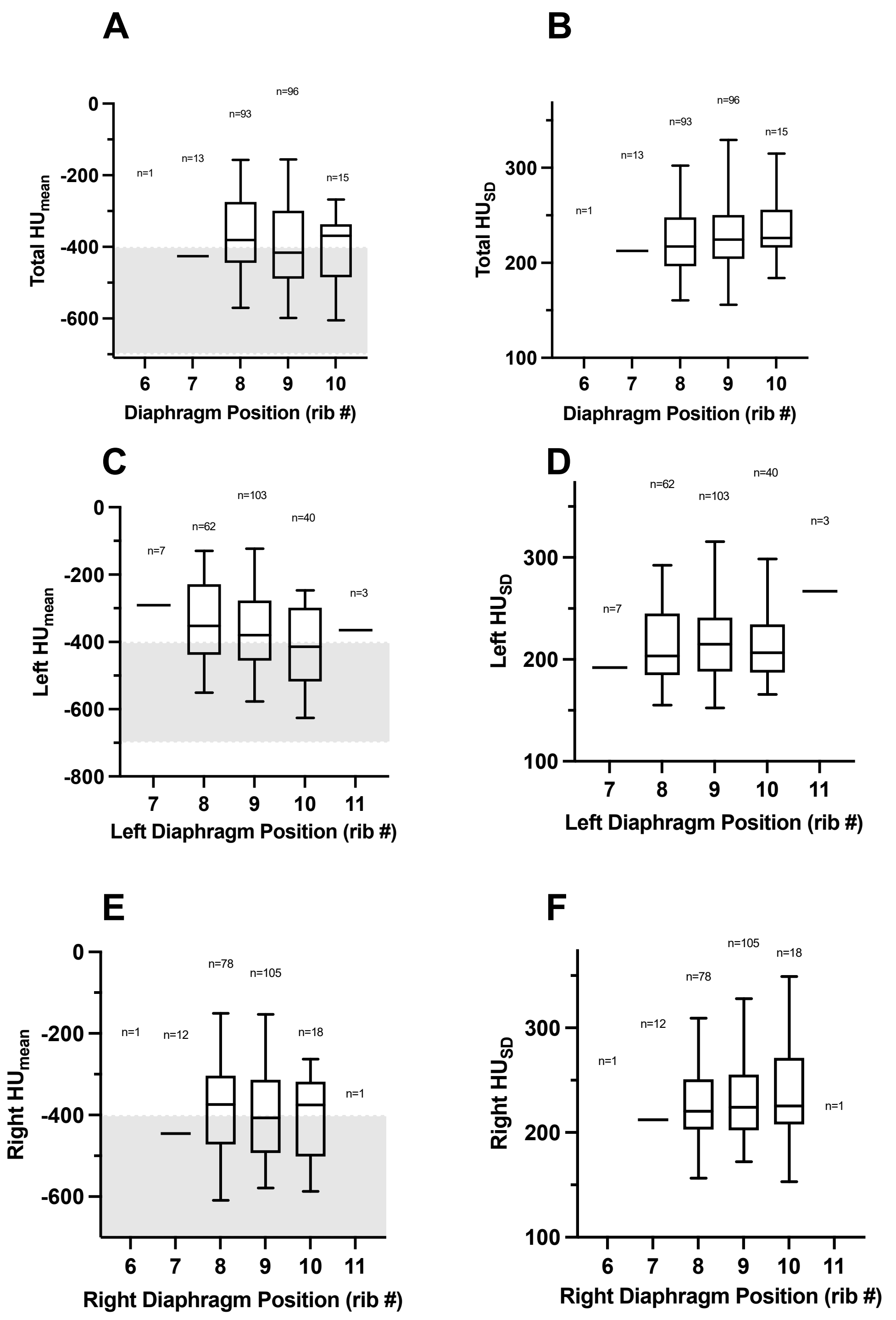

eFigure 15. Relationship between HU and diaphragm position (rib #) for the total HU_{mean} (A) and HU_{SD} (B) where the smaller rib number was used if the left and right diaphragm position differed. **Left lung HU_{mean} (C) and HU_{SD} (D), and right lung HU_{mean} (E) and HU_{SD} (F)** using the greater rib number when the left and right diaphragm position differed. Symbols and descriptors as per eFigure 10. Grey shading represents the normal HU range for lung tissue (-440, -700 HU).[4] Kendal Tau's correlation coefficient; (A) τ = -0.07 (-0.17,0.2), (B) τ = 0.1 (0.00,0.2), (C) τ = -0.14 (-0.23,-0.03), (D) τ = 0.08 (-0.03,0.18), (E) τ = -0.02 (-0.13,0.08), (F) τ = 0.07 (-0.04,0.17).

© 2025 Dahm SI et al. *JAMA Pediatrics*.

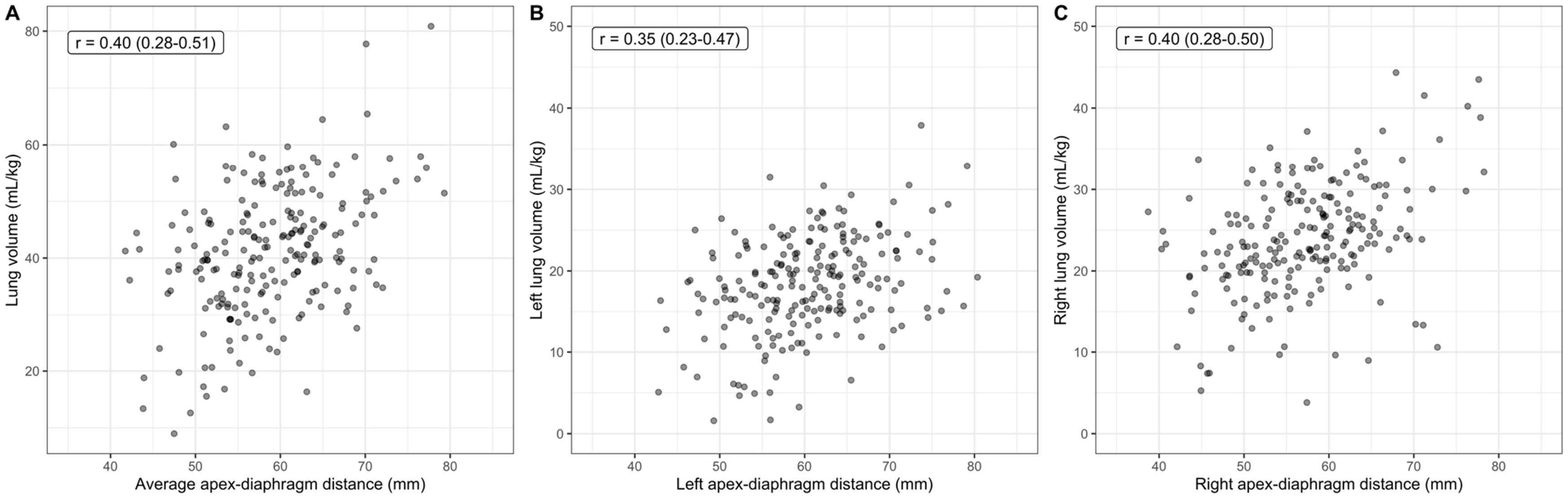

eFigure 16. Total (A) and Left (B) and Right (C) V_L (mL/kg) compared with respective apex-diaphragm distance (mm). Black circles represent individual infants. Pearson correlation (r) and 95% CI shown in each figure.

© 2025 Dahm SI et al. *JAMA Pediatrics*.
