# Supplementary material for: Diaphragm Position on Chest Radiograph to Estimate Lung Volume in Neonates
Source: JAMA Pediatr. 2025 Jul 21;179(9):1034–42. doi: 10.1001/jamapediatrics.2025.2108 (PMC12281396; doi:10.1001/jamapediatrics.2025.2108)
Supplement: Supplement 2. — Data Sharing Statement. [file jamapediatr-e252108-s002.pdf]

# Data Sharing Statement

Dahm. Diaphragm Position on Chest Radiograph to Estimate Lung Volume in Neonates. *JAMA Pediatr*. Published July 21, 2025. doi:10.1001/jamapediatrics.2025.2108

**Data**
**Data available:** Yes
**Data types:** Deidentified participant data, Data dictionary
**How to access data:** All data, including raw data used for all figures and analysis and data dictionaries, is available upon request to the corresponding author from three months following article publication to researchers who provide a methodologically sound proposal, with approval by an independent review committee ("learned intermediary"). Proposals should be directed to david.tingay@mcri.edu.au to gain access. Data requestors will need to sign a data access or material transfer agreement approved by Royal Children's Hospital and/or Murdoch Children's Research Institute.
**When available:** With publication

**Supporting Documents**
**Document types:** None

**Additional Information**
**Who can access the data:** Researchers who provide a methodologically sound proposal, with approval by an independent review committee ("learned intermediary")
**Types of analyses:** For any methodologically sound proposals
**Mechanisms of data availability:** Approval by an independent review committee ("learned intermediary")
